# Antiviral and Immune Enhancement Effect of *Platycodon grandiflorus* in Viral Diseases: A Potential Broad-Spectrum Antiviral Drug

**DOI:** 10.3390/molecules30040831

**Published:** 2025-02-11

**Authors:** Pei Gao, Xinshan Li, Jianlei Ding, Bosen Peng, Muhammad Munir, Fei Liu, Limin Chao, Chengfei Li, Li Wang, Jinyou Ma, Gaiping Zhang

**Affiliations:** 1 Postdoctoral Research Station, Henan Agriculture University, Zhengzhou 450002, China; gaopei@hist.edu.cn; 2 Postdoctoral Research Base, Henan Institute of Science and Technology, Xinxiang 453003, China; 3 College of Animal Science and Veterinary Medicine, Henan Institute of Science and Technology, Xinxiang 453003, China; 4 Henan International Joint Laboratory of Animal Health Breeding and Disease Prevention and Control, Xinxiang 453003, China; 5 Ministry of Education Key Laboratory for Animal Pathogens and Biosafety, Zhengzhou 450002, China; 6 Division of Biomedical and Life Sciences, Faculty of Health and Medicine, Lancaster University, Lancaster LA14YW, UK; 7 School of Advanced Agricultural Science, Peking University, Beijing 100871, China

**Keywords:** *Platycodon grandiflorus*, traditional Chinese medicine, respiratory infections, antiviral activity, inflammation suppression, immunomodulation

## Abstract

Background: Traditional Chinese medicine offers potential therapeutic options for viral infections. *Platycodon grandiflorus* (PG) is a perennial herb known for its efficacy in treating respiratory infections, including asthma, cough, and bronchitis, making it a key focus in antiviral drug research. The purpose of the study is to provide a basis for functional studies on PG and generate new insights for treating viral diseases. Methods: Research articles from 1990 to 2024 related to PG and viruses were obtained from databases, such as PubMed, Web of Science, and Science Direct, and systematically analysed. Results: PG demonstrates inhibitory effects on viruses such as severe acute respiratory syndrome coronavirus and porcine reproductive and respiratory syndrome virus by blocking various stages of viral proliferation or activating the host immune system. It also reduces inflammation through NF-κB, PI3K/AKT, MAPK, and other signalling pathways, enhancing T cell and macrophage function and increasing host immunity. PG exhibits diverse pharmacological effects with promising clinical applications for antiviral and immune modulation. Given its medicinal significance, PG holds substantial potential for further exploration and development. Conclusion: PG, due to its antiviral, anti-inflammatory, and immune-boosting properties, can be used as an antiviral drug.

## 1. Introduction

Viruses are microbial particles consisting of proteins and nucleic acids that cannot survive independently and must reside within host cells to replicate. They can infect humans, animals, microorganisms, and other organisms, causing various diseases, including respiratory, intestinal, and skin conditions [1,2,3,4]. In humans and animals, viral infection can lead to a spectrum of symptoms, ranging from asymptomatic to severe illness [5,6]. Viral replication requires the invasion of living cells; once inside, viruses release their nucleic acid, which serves as a template for replicating progeny viruses [7,8]. Characterised by high variability and rapid spread, viruses hijack host cells and cause substantial harm. Vaccines and immunization are the primary methods for preventing viral diseases. However, due to strain variation and limitations in vaccine efficacy, few vaccines provide complete protection. Many viral infections are also resistant to effective treatment with antiviral drugs. Therefore, the prevention and control of viral infections remain challenging.

Traditional Chinese medicine (TCM) boasts a rich history of employing plant-derived materials for therapeutic purposes. Many TCM herbs, sourced from plant foods, contain a myriad of phytochemicals with antiviral potential. Various medicinally active chemical compounds, such as polyphenols, polysaccharides, and flavonoids, can be extracted from plants. These phytochemicals exhibit significant antiviral activities and serve as a powerful arsenal against many viral diseases [9]. For example, natural polyphenols exert their therapeutic impacts on influenza infection through multiple cellular and molecular mechanisms. They can suppress the activity of neuraminidase (NA) and hemagglutinin (HA), disrupt the virus replication cycle, inhibit viral hemagglutination, block viral adhesion to and penetration into host cells, and interfere with intracellular transduction signalling pathways [10]. Purified fucose-rich polysaccharides demonstrate a wide-spectrum antiviral effect against both DNA and RNA viruses, including hepatitis C virus (HCV), adenovirus 7, and human immunodeficiency virus (HIV) [11]. Previous studies have provided conclusive evidence that phytochemicals possess antiviral potential. The emergence of coronavirus disease 2019 (COVID-19) has impelled researchers to concentrate on respiratory viral diseases. Among herbs, *Platycodon grandiflorus* (PG) has garnered significant attention due to its efficacy in alleviating lung inflammation and its antiviral capabilities.

*Platycodon grandiflorus* is a traditional herb widely used in traditional Chinese medicine. The roots of the plant are widely documented and primarily utilized for the treatment of respiratory ailments such as cough, sore throat, and phlegm [12,13,14,15]. PG also demonstrates anti-inflammatory, antibacterial, antiviral, immunomodulatory, and antitumour properties [16,17,18,19,20]. Advances in modern science and technology have enabled research on PG to uncover various bioactive components and their potential mechanisms of action, enhancing our understanding of its pharmacological effects and broadening its clinical applications. The primary active components of PG are platycodin, polysaccharides, flavonoids, and volatile oils [21,22,23]. PG is recognized for its efficacy in treating respiratory diseases by aiding lung function, soothing the throat, and expelling phlegm. Consequently, PG is often used for managing acute and chronic coughs, bronchitis, pharyngitis, and related respiratory conditions. Modern pharmacological studies suggest that PG can alleviate coughing and promote sputum clearance by enhancing tracheal mucociliary movement, thereby reducing airway resistance [24,25]. Additionally, PG exhibits notable antibacterial and antiviral activities, which help prevent respiratory infections caused by pathogens [26,27].

The anti-inflammatory and immunomodulatory activities of PG have garnered considerable interest in recent years [28,29,30,31]. Inflammation serves as a host defence mechanism against injury and infection. However, excessive or chronic inflammation can lead to tissue damage and disease. Studies indicate that Platycodon saponins can inhibit the expression of inflammatory mediators, thereby reducing inflammation [32]. Additionally, Platycodin D (PD) has been shown to enhance immune defence in mice by increasing serum antigen-specific antibody titres, promoting transcription factor and Th1/Th2 cytokine expression in spleen cells, and enhancing the cytotoxic activity of natural killer (NK) cells [33]. These findings highlight PG as a promising candidate for treating immune-related diseases and inflammatory conditions. This article reviews current research on the role of PG in preventing and treating viral diseases to provide a comprehensive overview of scientific progress and suggest directions for future research on PG in preventive and therapeutic applications. These findings highlight PG as a promising candidate for treating immune-related diseases and inflammatory conditions. However, the role of PG in virus-associated host response lacks systematic review.

In order to achieve a more all-encompassing and profound comprehension of the potential of PG as an antiviral agent, this article reviews current research on the role of PG in preventing and treating viral diseases in three ways: (1) it updates the recently described chemical composition of PG to provide a reference for network pharmacological analysis and drug functional studies; (2) it summarizes the mechanisms of action involved in cellular states, inflammation, and immunity, which will help us understand why PG works against a variety of viruses as well as provide a reference for innovative drug development; and (3) it presents the effects of PG combined with other herbs, which provides ideas for optimizing the antiviral effect of drugs.

## 2. Traditional Medicinal Uses

PG is the dried root of the Campanulaceae plant *Platycodon grandiflorus* (Jacq) A. DC. The initial documentation of PG can be found in the Agriculture God’s Canon of Materia Medica, which describes PG as an effective remedy for alleviating cough and asthma. The *Chinese Pharmacopoeia* lists it as treatment for cough and phlegm, chest tightness, sore throat, hoarseness, and abscess [34]. Known as the “boat of various medicines”, PG is traditionally valued for its ability to deliver other medicinal substances to different parts of the body while also promoting drainage and relief from ailments. PG has a longstanding history of application as both a food and medicinal herb. Since the Eastern Han Dynasty, the medicinal properties of PG have been continually revised and expanded. Its flavour has evolved from a single taste, “Xin” (pungent), to a compound taste, “bitter and Xin”, while its associated meridians have changed from “hand Taiyin”, “hand Shaoyin”, and “foot Yangming stomach” to later associations with the lung, heart, and stomach meridians, and finally with the lung meridian. PG is a frequent component in numerous prescriptions for treating lung diseases, such as Platycodon decoction, Ningfei Zhike powder, and Jiawei Qingzhong powder.

## 3. Chemical Composition of *Platycodon grandiflorus*

The chemical composition of PG is diverse and complex. Researchers have identified 229 chemical constituents in PG that encompass 88 saponins (Table 1) [35,36], 5 sterols (Table 2), 18 flavonoids (Table 3), 7 triterpenes (Table 4), 16 phenolic acids (Table 5), 7 polyacetylenes (Table 6), 34 fatty acids [37], 22 trace elements [38], 21 polysaccharides [39], and 17 amino acids [40]. Saponins mainly exist in the roots of PG, whereas flavonoids exist in the aerial parts. Active ingredients present in the roots have better pharmacological activities and have been well-studied in general. However, due to the limitations of instrument analysis, the active components of PG have not been completely analyzed. Among these functions, the antiviral activity of PG is gradually receiving attention.

## 4. Inhibitory Effects of *Platycodon grandiflorus* on Viruses

PG, a natural Chinese herbal medicine, has demonstrated considerable inhibitory effects against viral diseases. Six active triterpenoid saponins—platyconic acid A, Platycodin D, Platycodin D2, Platycodin D3, deapioplatycodin D, deapioplatycodin D2—and a PG saponin mixture have shown anti-hepatitis C virus (HCV) activity. Notably, PG saponin mixture exhibits strong anti-HCV effects when combined with either NS5A inhibitors or interferon-α [72]. The transient receptor potential anchor protein type 1 (TRPA1), an injury receptor activated by tissue damage and inflammation, is implicated in coronavirus disease 2019 (COVID-19) and serves as an important target of PG [73,74,75].

PD markedly suppresses the replication of the porcine reproductive and respiratory syndrome virus (PRRSV) by directly adhering to virions, subsequently influencing multiple phases within the viral life cycle. These phases encompass RNA synthesis, protein expression, and the release of progeny viruses [76]. PD is also predicted to be a potential inhibitor of papain-like proteases in severe acute respiratory syndrome coronavirus 2 (SARS-CoV-2) [77], effectively blocking two main infection pathways of SARS-CoV-2, which are mediated by transmembrane protease serine 2 (TMPRSS2) and lysosome-driven entry [78]. PG polysaccharide (PGPS_t_) inhibits Pseudorabies virus (PRV) replication by activating the Akt/mTOR pathway and suppressing autophagy [79]. BC703, a hot water extract of PG, inhibits HCV RNA replication and offers substantial hepatoprotection against acute hepatic injury caused by carbon tetrachloride (CCl_4_), as it reduces serum enzyme levels, nitric oxide, and lipid peroxidation [80]. Additionally, phytochemicals from PG target TMPRSS2 and disrupt the entry process of SARS-CoV-2 [81] (Table 7).

## 5. *Platycodon grandiflorus* Influences Viral Proliferation by Regulating Cellular States

PG regulates the cell state mainly through PGPS_t_. PGPS_t_ upregulates the expression level of the anti-apoptotic protein Bcl-2 while downregulating that of the pro-apoptotic protein Bax, thereby protecting against PRV-induced apoptosis. This protective effect is achieved by mitigating mitochondrial membrane potential decline, apoptosis, and structural damage, such as mitochondrial swelling, membrane thickening, and cristae disruption [82]. PGPS_t_ is also capable of mitigating respiratory syncytial virus-induced epithelial cell apoptosis. This effect is achieved via the activation of the miR-181a-regulated Hippo and SIRT1 signaling pathways [25]. Additionally, PGPS_t_ inhibits PRV-induced autophagosome accumulation via the Akt/mTOR signalling pathway [79]. Treatment with PGPS_t_ enhances LC3 colocalisation with SOCS1 and SOCS2, significantly affecting autophagy processes [83]. PGPS_t_ promotes major histocompatibility complex class I/II, CD40, CD80, and CD86 expression on cell surfaces, signifying the phenotypic maturation of DCs. Furthermore, PGPS_t_ increases the production of IL-1b, IL-6, IL-10, IL-12, tumour necrosis factor-a, and interferon (IFN)-β, indicating functional DC maturation. The induction of DC maturation by PG involves activation of the MAPK and NF-κB signalling pathways downstream of TLR4 [84]. In macrophages, PGPS_t_ stimulates NO and iNOS expression via the TLR4/NF-κB pathway [85]. Additionally, PD markedly suppresses tumour growth by enhancing immune function, inducing apoptosis, and inhibiting angiogenesis [86]. The aqueous extract of PG exerts a promotive influence on the proliferation, spreading, phagocytic activity, and cytostatic function of macrophages, as well as the generation of nitric oxide, while elevating inflammatory marker levels, highlighting the potent stimulatory effects of PG on macrophages [87].

## 6. *Platycodon grandiflorus* Affects Disease Progress by Suppressing Inflammation

**Figure 1:**
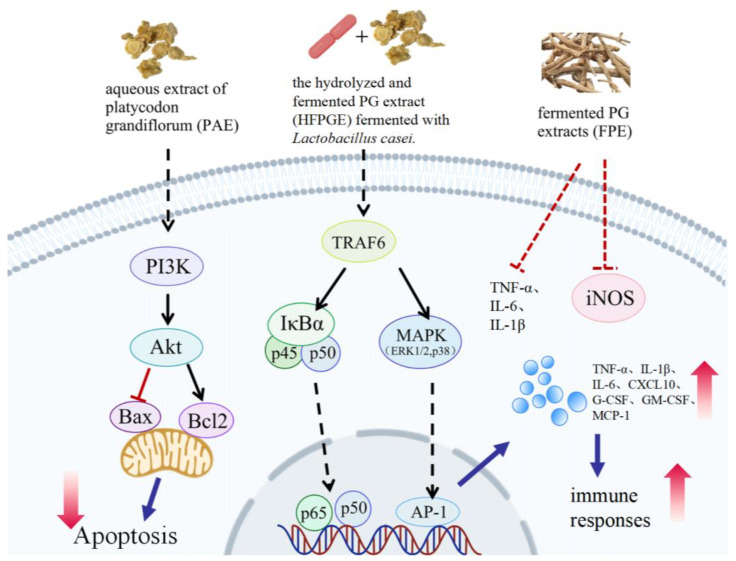
*Platycodon grandiflorus* affects cell states and inflammation through different signaling pathways. The figure was created using Adobe Illustrator 2024 (64 bit). Abbreviation: PI3K: Phosphoinositide 3-kinase; Protein kinase B is also known as Akt; Bcl2: B-cell lymphoma-2; Bax: BCL2-Associated X; TRAF6: TNF receptor associated factor 6; Nuclear factor of kappa light polypeptide gene enhancer in B-cells inhibitor alpha is also known as IκBα; MAPK: Mitogen-activated protein kinase; ERK: Extracellular regulated protein kinases; AP-1: Activator protein 1; TNF: Tumor necrosis factor; IL: Interleukin; CXCL: Chemokine (C-X-C motif) ligand; G-CSF: Granulocyte colony-stimulating factor; GM-CSF: Granulocyte-macrophage colony-stimulating factor; MCP-1: Monocyte Chemoattractant Protein-1.

PG is a potential anti-inflammatory agent, exerting its effects via multiple signalling pathways [88,89,90]. PG root extracts promote the microglial phagocytosis of Aβ and reduce Aβ deposition and neuroinflammation, preventing neuronal cell death in Alzheimer disease [91]. PG root extract reduces the inflammatory response to acute lung injury by preventing apoptosis via the PI3K/Akt signalling pathway [92]. Fermented PG extracts suppress iNOS and several proinflammatory cytokines, thereby reducing airway inflammation [32]. However, after fermentation with *Lactobacillus casei*, the abundances of crude saponin and PD increase, and hydrolysed and fermented PG extracts boost the yield of TNF-α, IL-1β, tC-X-C motif chemokine ligand 10, and granulocyte colony-stimulating factor through activation of the MAPK and NF-κB signalling pathways [93] (Figure 1). This variation may be related to differences in active ingredient content. Appropriate doses of PG inhibit inflammatory responses, while excessive doses may produce opposing effects. PGPS_t_ can act synergistically with PD to alleviate excessive mucus secretion, histopathological abnormalities, and immune imbalance in rat lungs, which are closely linked to small intestinal mucosal immunity. This finding provides a novel perspective supporting the traditional theoretical construct of TCM in the lungs and intestine [94].

**Figure 2:**
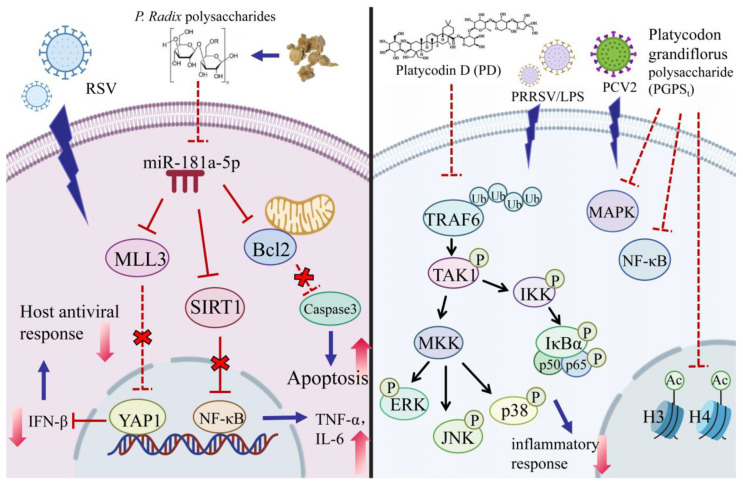
Effects of different active components of *Platycodon grandiflorus* on inflammation in different cell lines. The figure was created using Adobe Illustrator 2024 (64 bit). Abbreviation: RSV: Respiratory syncytial virus; miR: microRNA; MLL: Myeloid/lymphoid or mixed-lineage leukemia; SIRT1: Sirtuin; Bcl2: B-cell lymphoma-2; NF-κB: Nuclear factor kappa- light- chain- enhancer of activated B cells; YAP1: Yes-associated protein 1; TNF: Tumor necrosis factor; IL: Interleukin; IFN: Interferon; PRRSV: Porcine Reproductive and Respiratory Syndrome virus; LPS: Lipopolysaccharides; TRAF6: TNF receptor associated factor 6; Ub: Ubiquitin; TAK1: Transforming Growth Factor-β-Activated Kinase 1; MKK: Mitogen-Activated Protein Kinase Kinase; ERK: Extracellular regulated protein kinases; JNK: c-Jun N-terminal kinase; IKK: Inhibitor of kappa B kinase; Nuclear factor of kappa light polypeptide gene enhancer in B-cells inhibitor alpha is also known as IκBα; MAPK: Mitogen-activated protein kinase; Ac: Acetyl.

PD decreases PRRSV-induced and lipopolysaccharide-induced cytokine inflammatory factor production in primary porcine alveolar macrophages [76]. PD binds to TRAF6, reducing its K63 ubiquitination and inhibiting the activation of the MAPK and TAK1/IKK/NF-κB pathways, downregulating the overactivated inflammatory response and immune cell infiltration, which improves the survival rate of influenza [95]. PGPS_t_ can mitigate the inflammatory factor expression caused by porcine circovirus type 2 through the modulation of histone acetylation and inhibiting the activation of NF-κB and MAPK signaling pathways, thereby reducing the release of inflammatory factors and pro-inflammatory enzymes [27]. PGPS_t_ improves respiratory syncytial virus-induced inflammation through Hippo and SIRT1 pathways, which is mediated by miR-181a [25] (Figure 2).

The processing method of *Platycodonis radix* may affect the effects of PG. Baihezhijiegeng is derived from processed *Platycodonis radix*. Baihezhijiegeng administration significantly decreases IL-1β, IL-6, TNF-α, and matrix metalloproteinase 9 expression; decreases IFN-γ levels; increases IL-4 and IL-10 expression; and improves the pathological condition of the lungs in rats with chronic obstructive pulmonary disease [96]. Fermented PG extract increases NF-κB levels and modulates the expression of NO and proinflammatory cytokines [97].

## 7. *Platycodon grandiflorus* Affects Disease Progress by Enhancing Immunity

PG enhances viral immunogenicity by means of both cellular and humoral immune responses. PG extracts increase the production of cyclophosphamide-induced immunoglobulins (IgG and IgA) and inflammatory cytokines in splenocytes and serum, enhance the activity of cytotoxic T lymphocytes and NK cells, and help recover white blood cell, lymphocyte, and neutrophil counts [98]. PG modulates gut microbiome abundance by regulating IgA and IgM levels, indicating that dietary PG can improve the health status of mice with suppressed immune systems [99]. A clinical study indicated that IFN-γ levels and NK cell activity were elevated in a PG-treated group compared with a placebo group, with no significant clinical changes or serious adverse events reported [100]. The immune function of macrophages can be stimulated using different polysaccharide extraction methods, such as hot water (PG-H), ultrasonic-assisted (PG-U), and acid-assisted (PG-C) extraction; among these, PGs with higher GalA content and lower molecular weights showed superior immune-stimulating activity [101].

PG or fermentation by *Lactobacillus plantarum* could increase Th1 cytokines IL-12p40 and IFN-γ while reducing Th2 cytokines IL-4 and IL-5, thus preventing the progression of atopic dermatitis-like skin lesions [13,102]. Hydrolysed and fermented PG extracts restore serum levels of IgA, IgM, IgG, IL-12, IL-8, TNF-α, and transforming growth factor (TGF)-β reduced by CPA treatment, while increasing splenocyte proliferation and splenocyte IL-8, IL-4, and TGF-β levels [103].

PGPS_t_ brings about an augmentation in the quantity of CD4+ and CD8+ T cells and simultaneously facilitates lymphocyte cycle progression from the G0/G1 phase to the S phase and G2/M phase [104]. PGPS_t_ regulates colonic immunity by restoring the levels of Th1, Th2, Th17, transcription factors, and Treg-related cytokines in the colon via regulating the equipoise of colonic immune cells and thereby regulating colonic immunity to remit DSS-induced ulcerative colitis (UC) [105]. Other studies in mice suggested that PGPS_t_ activates NO production and iNOS transcription in macrophages and increases B cell proliferation and polyclonal IgM antibody production but does not affect T cell proliferation and IL-2 or IL-4 expression in Th1 and Th2 cells, suggesting that PG is distinct from other immunostimulants [106].

PG cooperates with *Salvia plebeian* extracts and activates macrophages through the MAPK and NF-κB signalling pathways to stimulate the production of IL-1β, IL-6, PGE2, COX-2, and TNF-α [107]. The adjuvant effects of PG saponins on OVA-specific IgG2b, IgG, and IgG1 levels in mice are notably more pronounced compared to those exerted by alum [108]. Platycodin D, D3, and D2 significantly promote nonspecific immunity in the serum, and only platycoside E (PE) significantly promotes the production of IgG2a and IgG2b antibodies in OVA-immunised mice [109]. Platycodin D treatment significantly increases the anti-IB antibody titre and chicken peripheral blood mono-nuclear cell proliferation and expression, resulting in a lower mortality rate, fewer and less severe clinical signs, and no observed side effects [110]. PD and PD2 elevate serum titres of HBsAg-specific antibodies and enhance the production of Th1 and Th2 cytokines in splenocytes [111]. PD markedly increases the killing activity of CTLs and NK cells by splenocytes in HBsAg-immunised mice [112].

Platycodin D significantly promotes lipopolysaccharide concanavalin A. It promotes antigen-induced splenocyte proliferation and augments the production of serum antigen-specific antibody titres in mice immunised with rL-H5. Platycodin D also increases the killing activity of splenocyte NK cells [33]. When comparing the effects of PA, PD, PD2, PD3, PE, deapioplatycoside E, and polygalacin D2 from PG on the immune response to the Newcastle disease virus-based recombinant avian influenza vaccine (rL-H5) in mice, researchers found that PD and PD2 increased antigen-specific antibody titres. They also found that their biological and adjuvant activities were affected by the sugar chains at C-3, the glycosidic group at C-28 of the aglycone, and the retention time as determined through reverse-phase HPLC analysis [113]. Among *Platycodon saponins*, PGS30, PGS50, PGS75, PGS95, PGS50, and PGS75 induce a balanced Th1/Th2 response to virus infection in mice, enhancing both cellular and humoral immune responses. Among these, PGS75 is a potential ideal adjuvant candidate for the hepatitis B vaccine [114].

In summary, both PD and PGPS_t_ enhance specific and nonspecific immunity, suggesting that PG can be used as an immune-enhancing agent to improve host disease resistance and vaccine immune effects.

## 8. Synergistic Effect of *Platycodon grandiflorus* with Other Drugs

TCM is extensively applied in the therapy of viral infections; however, the effects of individual TCM agents are generally mild, and combining these agents can enhance therapeutic and immunoregulatory benefits. PG serves as a “Yin-Jing” medicine targeting the lungs due to the active compounds, Platycodon saponins B and C [115]. PG is a component of various TCM prescriptions, including Qingjin Huatan decoction (QJHTT), Jie-Geng-Tang, Zhisou powder, and Ruyiping. Dietary supplementation with PG, *Panax ginseng*, *Atractylodes macrocephala*, *Dioscoreaceae*, *Glycyrrhiza uralensis*, and *Ziziphus* in pigs has been shown to significantly enhance Salmonella-killing capacity and respiratory burst activity [116]. QJHTT reduces lung tissue virus titres and improves the lung index, survival rate, and pulmonary histopathological changes. QJHTT effectively reduces levels of STAT3 and JAK2, further affecting the serum concentrations of IL-1β, TNF-α, IL-6, and IFN-γ, and reverses the activity of CCL2, CCL7, and CCR1 [117]. Additionally, QJHTT reduces influenza A virus titers and modulates the levels of inflammatory factors in lung tissue [118]. Jie-Geng-Tang may alleviate acute lung injury by modulating the MAPK and PI3K/Akt signaling pathways [119]. Zhisou powder inhibits activation of the PI3K/Akt/HIF-1α/VEGFA signaling pathway and reprograms arachidonic acid metabolism, contributing to its effectiveness in treating chronic bronchitis [120]. Ruyiping helps maintain microvascular integrity, reduces fibrinogen extravasation, and decreases the expression of CXCL2, CXCL5, IL-1β, and IL-6 [121].

## 9. Conclusions and Perspective

**Figure 3:**
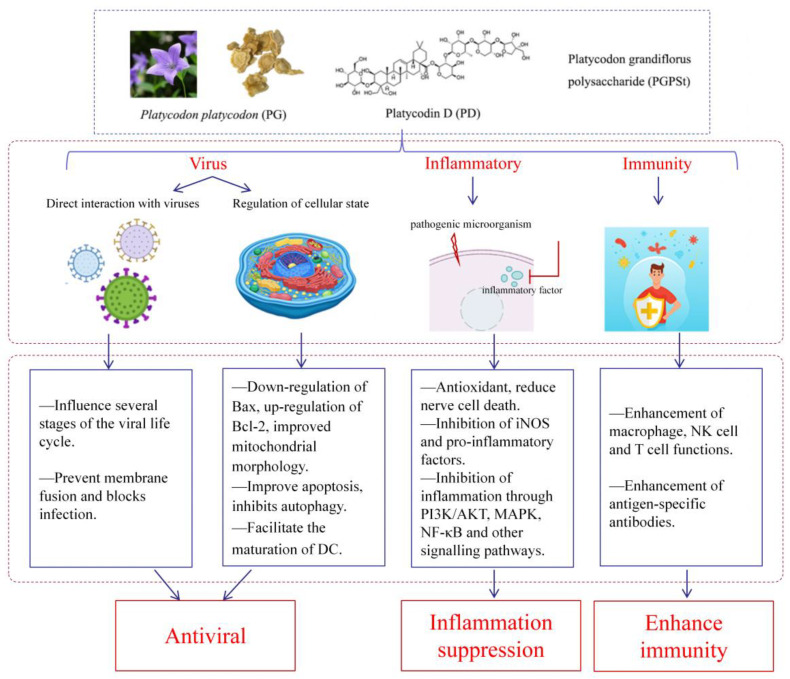
Generalization of the effects of *Platycodon grandiflorus.* The figure was created using Adobe Illustrator 2024 (64 bit). Abbreviation: Bcl2: B-cell lymphoma-2; Bax: BCL2-Associated X; DC: Dendritic cells; iNOS: Inducible nitric oxide synthase; PI3K: PhosphoInositide-3 Kinase; Protein kinase B is also known as Akt; MAPK: Mitogen-activated protein kinase; NF-κB: Nuclear factor kappa- light- chain- enhancer of activated B cells; NK cell: Natural Killer cell; T cell: T lymphocyte.

PG is employed as a traditional Chinese medicine due to its antiviral, immune-enhancing, and anti-inflammatory characteristics that inhibit various stages of viral proliferation. PG activates the host immune system to inhibit viruses such as SARS, PRV, and PRRSV and suppresses inflammation through PI3K/AKT, MAPK, NF-κB, and other signaling pathways. Additionally, PG improves the function of macrophages, NK cells, and T cells and enhances both specific and non-specific immunity (Figure 3). These immune actions of PG demonstrate its significant potential for clinical applications.

The prophylaxis and management of viral diseases has become one of the key challenges that researchers are focusing on. Similar to the ongoing quest for a universal vaccine to prevent avian influenza virus [122], the exploration of universal antiviral drugs presents an alternative approach, and PG is one of the objects that can be considered. In this article, we concluded that PG contains as many as 229 active saponins, flavonoids, and other compounds, all of which have different functions. For example, these compounds may act on the virus directly to inhibit proliferation, or they could regulate the state of the host cell and clear the virus by downregulating the overactivated inflammatory response. Since the active ingredients inhibit the virus in different ways, drug resistance is unlikely to develop. In addition, PG can eliminate the virus by enhancing non-specific immunity. In this case, the immune system is activated against a variety of viruses. The combination of PG with other drugs further broadens the antiviral spectrum and enhances the effect. These characteristics indicate that PG holds great potential as a broad-spectrum antiviral agent and thus warrants further research and development.

Despite considerable advances in understanding pharmacological mechanisms of PG, several challenges remain. Primarily, the inhibitory effect of PG and its active ingredients need to be further developed and analysed. The complexity of the active ingredients in PG may mean that its efficacy is actually the outcome stemming from the synergistic interplay of multiple components, which makes it difficult to determine specific mechanisms of action [123]. In addition, existing studies have partially analysed the molecular mechanism underlying the antiviral activity of PG, but there are additional functions that call for further in-depth analysis. For example, PG root extract reduces the inflammatory response by preventing apoptosis via the PI3K/Akt signalling pathway [92], and PI3K can also participate in the regulation of autophagy, cell growth, and lipid synthesis [124,125,126]. In addition, TRAF, which is regulated by fermented PG extracts, may directly trigger the activation of PI3K [127]; thus, there is an intrinsic connection between them that is also worth exploring. Further research is also needed on the safety of long-term use of PG. Future studies should conduct systematic safety evaluations and clinical trials to clarify the active components and their mechanisms of action, thereby providing a scientific basis for the broader applications of PG.

## Figures and Tables

**Table 1:** Saponins in *Platycodon grandiflorus*.

No.	Compounds	Molecular Formula	Sources	Ref.
1	Platycodigenin	C_30_H_48_O_7_	Root	[41]
2	Platycodin C(3-O-Acetyl platycodin D)	C_59_H_94_O_29_	Root	[41]
3	Platycodin D	C_57_H_92_O_28_	Root	[42]
4	Platycodin D2	C_63_H_102_O_33_	Root	[43]
5	Platycodin D3	C_63_H_102_O_33_	Root	[42]
6	Platycodin J	C_57_H_90_O_29_	Root	[44]
7	Platycodin L	C_59_H_92_O_30_	Root	[44]
8	Deapi-platycodin D	C_52_H_84_O_24_	Root	[42]
9	Deapi-platycodin D2	C_58_H_94_O_29_	Root	[45]
10	Deapi-platycodin D3	C_58_H_94_O_29_	Root	[42]
11	Platycoside A	C_58_H_94_O_29_	Root	[46]
12	Platycoside B	C_54_H_86_O_25_	Root	[46]
13	Platycoside C	C_54_H_86_O_25_	Root	[46]
14	Platycoside E	C_69_H_112_O_38_	Root	[47]
15	Platycoside F	C_47_H_76_O_20_	Root	[48]
16	Platycoside G1(Deapi-platycoside E)	C_64_H_104_O_34_	Root	[49]
17	Platycoside G2	C_59_H_96_O_30_	Root	[49]
18	Platycoside I	C_64_H_104_O_33_	Root	[48]
19	Platycoside J	C_52_H_84_O_23_	Root	[48]
20	Platycoside K	C_42_H_68_O_17_	Root	[48]
21	Platycoside L	C_42_H_68_O_17_	Root	[48]
22	Platycoside P	C_53_H_56_O_25_	Root	[50]
23	β-Gentiotriosyl platycodigenin	C_48_H_78_0_22_	Root	[51]
24	3-O-β-D-Gentiotriosyl platycodigenin	C_36_H_58_O_12_	Root	[52]
25	3-O-β-D-Gentiotriosyl platycodigenin methyl ester	C_37_H_60_O_12_	Root	[53]
26	3-O-β-Gentiotriosyl platycodigenin methyl ester	C_43_H_70_O_17_	Root	[53]
27	3-O-β-Lentiotriosyl platycodigenin methyl ester	C_43_H_70_O_17_	Root	[53]
28	Platycoside D	C_69_H_112_O_37_	Root	[47]
29	Platycoside G3(Polygalacin D3)	C_63_H_102_O_32_	Root	[49]
30	Platycoside H	C_58_H_94_O_28_	Root	[48]
31	Platycoside N	C_53_H_86_O_24_	Root	[54]
32	Polygalacic acid	C_30_H_48_O_6_	Root	[41]
33	Polygalacin D	C_57_H_92_O_27_	Root	[42]
34	Polygalacin D2	C_63_H_102_O_32_	Root	[51]
35	Deapi-polygalacin D2	C_58_H_94_O_28_	Root	[55]
36	Deapi-polygalacin D3	C_58_H_94_O_28_	Root	[51]
37	-Gen-tiobiosy-platycodigenin	C_42_H_68_O_16_	Root	[51]
38	3-O-β-D-Glucopyranosyl polygalacic acid	C_36_H_58_O_11_	Root	[56]
39	3-O-β-D-Laminaribiosyl polygalacic acid	C_42_H_68_O_16_	Root	[56]
40	Methyl-3-O-β-B-D-glucopyranosyl polygalacate	C_37_H_60_O_11_	Root	[53]
41	Methyl-3-O-β-laminaribiosyl polygalacate	C_43_H_70_O_16_	Root	[53]
42	Platycogenic acid A	C_30_H_45_O_9_	Root	[41]
43	Platyconic acid A	C_57_H_90_O_29_	Root	[43]
44	Platyconic acid B	C_59_H_92_O_30_	Root	[44]
45	Platyconic acid C	C_52_H_82_O_25_	Root	[44]
46	Platyconic acid D	C_54_H_84_O_26_	Root	[44]
47	Platyconic acid E	C_58_H_92_O_30_	Root	[44]
48	Platycoside O	C_53_H_84_O_25_	Root	[54]
49	Platyconic acid A methyl ester	C_58_H_92_O_29_	Root	[43]
50	Methyl platyconate A	C_58_H_92_O_29_	Root	[53]
51	Methyl 2-O-methyl platyconate A	C_58_H_94_O_29_	Root	[53]
52	Dimethyl 2-O-methyl-3-O-β-D-glucopyranosyl platycogenate A	C_39_H_62_O_13_	Root	[53]
53	Dimethyl 3-O-β-D-glucopyranosyl platycogenate A	C_38_H_60_O_13_	Root	[53]
54	Platycoside Q	C_53_H_82_O_25_	Root	[50]
55	Platyconic acid A lactone	C_57_H_88_O_29_	Root	[43]
56	Platyconic acid B lactone	C_63_H_98_O_34_	Root	[45]
57	Deapi-platyconic acid A lactone	C_52_H_80_O_25_	Root	[43]
58	Deapi-platyconic acid B lactone	C_58_H_80_O_30_	Root	[45]
59	Platycogenic acid A lactone	C_30_H_44_O_8_	Root	[43]
60	O-β-D-Glucopyranosyl polatycogenic acid A lactone methyl ester	C_37_H_56_O_12_	Root	[53]
61	Platycodonoids A	C_29_H_45_O_5_	Root	[52]
62	Platycodonoids B	C_35_H_56_O_10_	Root	[52]
63	16-Oxo-platycodin D	C_57_H_90_O_28_	Root	[57]
64	Platycodsaponin A	C_42_H_68_O_16_	Root	[44]
65	Platycogenic acid B	C_30_H_46_O_9_	Root	[41]
66	Platycogenic acid C	C_30_H_45_O_6_	Root	[41]
67	3-O-β-D-Glucopyranosyl-2,12x,16x,23,24-pentahydroxy-oleanane-28(13)-lactone	C_36_H_58_O_13_	Root	[58]
68	Platycodon A	C_42_H_68_O_16_	Root	[59]
69	Platycodon B	C_41_H_66_O_15_	Root	[59]
70	3-O-β-D-Glucopyranosyl-(1 → 3)- β-D-glucopyranosyl-2β,12α,16α,23α-tetrahydroxy-oleanane- 28(13)-lactone	C_42_H_68_O_17_	Root	[58]
71	Deapi-3″-O-acetyl platycodin D	C_54_H_86_O_25_	Root	[55]
72	Platycoside M-3	C_52_H_80_O_24_	Root	[48]
73	Deapi-2″-O-acetyl platycodin D2	C_60_H_96_O_30_	Root	[51]
74	Deapi-2″-O-acetyl polygalacin D2	C_60_H_95_O_30_	Root	[55]
75	Deapi-2″-O-acetyl polygalacin D3	C_60_H_95_O_30_	Root	[55]
76	Dexyl-2″-O-acetyl polygalacin D3	C_55_H_87_O_25_	Root	[55]
77	Platycoside M-2	C_47_H_72_O_20_	Root	[48]
78	Platycoside M-1	C_36_H_54_O_12_	Root	[48]
79	2′-O-Acetyl platycodin D2	C_65_H_104_O_34_	Root	[51]
80	2′-O-Acetyl platycodin D3	C_65_H_104_O_34_	Root	[51]
81	2″-O-Acetyl polygalacin D	C_59_H_94_O_28_	Root	[42]
82	2″-O-Acetyl polygalacin D2	C_65_H_104_O_33_	Root	[45]
83	Platycodin A (2″-O-Acetyl platycodin D)	C_59_H_94_O_29_	Root	[41]
84	3′-O-Acetyl platycodin D2	C_65_H_104_O_34_	Root	[55]
85	3′-O-Acetyl platycodin D3	C_65_H_104_O_34_	Root	[51]
86	3″-O-Acetyl polygalacin D	C_59_H_94_O_28_	Root	[42]
87	3″-O-Acetyl polygalacin D2	C_65_H_104_O_33_	Root	[45]
88	3″-O-Acetyl polygalacin D3	C_65_H_104_O_34_	Root	[55]

**Table 2:** Sterols in *Platycodon grandiflorus*.

No.	Compounds	Molecular Formula	Sources	Ref.
1	δ-7-stigmastenone-3	C_29_H_46_O	Root	[60]
2	β-sitosterol	C_29_H_50_O	Root	[60]
3	α-spinasteryl-3-O-β-D-glucoside	C_35_H_60_O_6_	Root	[60]
4	spinasterol	C_29_H_48_O	Root	[60]
5	betulin	C_30_H_50_O_2_	Root	[60]

**Table 3:** Flavonoids in *Platycodon grandiflorus*.

No.	Compounds	Molecular Formula	Sources	Ref.
1	(2R, 3R)-Taxifolin	C_15_H_12_O_7_	Seeds	[61]
2	Apigenin	C_15_H_10_O_5_	Aerial parts	[62]
3	Apigenin 7-O-β-D-glucopyranoside	C_21_H_20_O_10_	Aerial Parts	[63]
4	Apigenin-7-0-glucoside	C_21_H_20_O_10_	Aerial parts	[62]
5	Delphinidin-3-rutinoside-7-glucoside	C_33_H_42_O_16_	Flowers	[64]
6	Dorajiside Ⅱ	C_27_H_36_O_14_	Aerial Parts	[63]
7	Dorajiside I	C_26_H_34_O_14_	Aerial Parts	[63]
8	Flavoplatycoside	C_27_H_32_O_16_	Seeds	[61]
9	Lonicerin	C_27_H_30_O_15_	Aerial Parts	[63]
10	Luteolin	C_15_H_10_O_7_	Aerial parts	[62]
11	Luteolin 7-O-(6″-O-acetyl)-β-D-glucopyranoside	C_23_H_22_O_12_	Aerial Parts	[63]
12	Luteolin 7-O-β-D-glucopyranoside	C_21_H_20_O_11_	Aerial Parts	[63]
13	Luteolin-7-0-glucoside	C_21_H_20_O_11_	Seeds, aerial parts	[61]
14	Platyconin	C_63_H_74_O_37_	Flowers	[61]
15	Platycoside	C_20_H_20_O_7_	Seeds	[61]
16	Quercetin-7-0-glucoside	C_21_H_20_O_12_	Seeds	[61]
17	Quercetin-7-0-rutinoside	C_27_H_30_O_16_	Seeds	[61]
18	Rhoifolin	C_27_H_30_O_14_	Aerial Parts	[63]

**Table 4:** Triterpene in *Platycodon grandiflorus*.

No.	Compounds	Molecular Formula	Sources	Ref.
1	28-O-laurylbetulin	C_41_H_70_O_3_	Root	[65]
2	betulin	C_30_H_50_O_2_	Root	[65]
3	betulinaldehyde	C_30_H_48_O_2_	Root	[65]
4	lupeol	C_43_H_68_O_15_	Root	[65]
5	platycodonoid	C_30_H_50_O	Root	[65]
6	ursolic acid	C_30_H_48_O_3_	Root	[65]
7	β-D-glucopyranosyl-2α,3β, 23-trihydroxyolean-12-en-28-oate	C_36_H_58_O_10_	Root	[65]

**Table 5:** Phenolic acids in *Platycodon grandiflorus*.

No.	Compounds	Molecular Formula	Sources	Ref.
1	(+)-(7R,8R)-linoleyl alatusol D	C_26_H_40_O_4_	Root	[66]
2	(+)-(7R,8R)-palmitoyl alatusol D	C_24_H_40_O_4_	Root	[66]
3	2,3-dihydroxybenzoic acid	C_7_H_6_O_4_	Aerial Parts	[62]
4	2-hydroxy-4-methoxybenzoic acid	C_8_H_8_O_4_	Aerial Parts	[62]
5	3,4-dimethoxycinnamic acid	C_11_H_12_O_4_	Aerial Parts	[62]
6	caffeic acid	C_9_H_8_O_4_	Aerial Parts	[62]
7	chlorogenic acid	C_16_H_18_O_9_	Aerial Parts	[62]
8	coniferyl oleate	C_27_H_40_O_4_	Root	[67]
9	coniferyl palmitate	C_25_H_38_O_3_	Root	[67]
10	coumaric acid	C_9_H_8_O_3_	Aerial Parts	[62]
11	ferulic acid	C_10_H_10_O_4_	Aerial Parts	[62]
12	homovanillic acid	C_9_H_10_O_4_	Aerial Parts	[62]
13	isoferulic acid	C_10_H_10_O_4_	Aerial Parts	[62]
14	m-coumaric acid	C_9_H_8_O_3_	Aerial Parts	[62]
15	p-hydroxybenzoic acid	C_7_H_6_O_3_	Aerial Parts	[62]
16	α-resorcylic acid	C_7_H_6_O_4_	Aerial Parts	[62]

**Table 6:** Polyacetylene in *Platycodon grandiflorus*.

No.	Compounds	Molecular Formula	Sources	Ref.
1	Cordifolioidyne C	C_17_H_24_O_6_	flowers	[68]
2	isolobetyol	C_9_H_8_O_4_	root	[69]
3	lobetyol	C_11_H_18_O_3_	root	[70]
4	lobetyolin	C_17_H_30_O_9_	root	[70]
5	lobetyolinin	C_23_H_40_O_14_	root	[70]
6	platetyolin A	C_17_H_30_O_9_	root	[71]
7	platetyolin B	C_17_H_30_O_9_	root	[71]

**Table 7:** Antiviral effect and molecular mechanism of *Platycodon grandiflorus*.

Active Constituent	Experimental Model	Doses/IC_50_	Virus	Effects	Ref.
Platycodin D	Marc-145 cells and primary porcine alveolar macrophages	1–4 µM	PRRSV	Interacts with virions and affects viral entry, PRRSV RNA synthesis, viral protein expression, progeny virus production, and progeny virus release.	[76]
Platycodin D	H1299 cells, Vero cells, Calu-3 cells	0.69 µM in H1299 cells, 4.76 µM in Vero and Calu-3 cells	SARS-CoV-2	Blocks TMPRSS2 and lysosome-driven entry	[77]
PGPS_t_	PK-15 cells	200 µg/mL	PRV	Activates the Akt/mammalian target of rapamycin (mTOR) pathway and inhibits autophagy	[79]
Hot water extract from PG	Mice	2.82 µg/mL	HCV	Inhibits HCV RNA replication and produces significant hepatoprotective effects against carbon tetrachloride (CCL4)-induced acute hepatic injury by reducing serum enzyme activities, nitric oxide concentrations, and the lipid peroxidation extent	[80]
Apigenin, Luteolin, Ferulic acid		3.33 µM, 10.39 µM, 13.95 µM	SARS-CoV-2	Targets the TMPRSS2 and disturbs the entry process	[81]

## Data Availability

Not applicable.

## Abbreviations

CCL4: carbon tetrachloride; COVID-19, coronavirus disease 2019; HCV, hepatitis C virus; IFN, interferon; IL, interleukin; JGT, Jie-Geng-Tang; mTOR, mammalian target of rapamycin; NF-κB, nuclear factor kappa B; NK, natural killer; PCV2, porcine circovirus type 2; PE, platycoside E; PG, *Platycodon grandiflorus*; PGPSt, *Platycodon grandiflorus* polysaccharide; PGSM, *Platycodon grandiflorus* saponin mixture; PRRSV, porcine reproductive and respiratory syndrome virus; PRV, Pseudorabies virus; QJHTT, Qingjin Huatan decoction; RP, Ruyiping; SARS-CoV-2, severe acute respiratory syndrome coronavirus 2; TCM, traditional Chinese medicine.; TGF, transforming growth factor; ZP, Zhisou powder.
